# Human Pituitary Adenoma Proteomics: New Progresses and Perspectives

**DOI:** 10.3389/fendo.2016.00054

**Published:** 2016-05-31

**Authors:** Xianquan Zhan, Xiaowei Wang, Tingting Cheng

**Affiliations:** ^1^Key Laboratory of Cancer Proteomics of Chinese Ministry of Health, Xiangya Hospital, Central South University, Changsha, China; ^2^Hunan Engineering Laboratory for Structural Biology and Drug Design, Xiangya Hospital, Central South University, Changsha, China; ^3^State Local Joint Engineering Laboratory for Anticancer Drugs, Xiangya Hospital, Central South University, Changsha, China; ^4^The State Key Laboratory of Medical Genetics, Central South University, Changsha, China

**Keywords:** pituitary adenoma, proteome, heterogeneity, reference map, two-dimensional gel electrophoresis, mass spectrometry, bioinformatics

## Abstract

Pituitary adenoma (PA) is a common intracranial neoplasm that impacts on human health through interfering hypothalamus–pituitary–target organ axis systems. The development of proteomics gives great promises in the clarification of molecular mechanisms of a PA and discovery of effective biomarkers for prediction, prevention, early-stage diagnosis, and treatment for a PA. A great progress in the field of PA proteomics has been made in the past 10 years, including (i) the use of laser-capture microdissection, (ii) proteomics analyses of functional PAs (such as prolactinoma), invasive and non-invasive non-functional pituitary adenomas (NFPAs), protein post-translational modifications such as phosphorylation and tyrosine nitration, NFPA heterogeneity, and hormone isoforms, (iii) the use of protein antibody array, (iv) serum proteomics and peptidomics, (v) the integration of proteomics and other omics data, and (vi) the proposal of multi-parameter systematic strategy for a PA. This review will summarize these progresses of proteomics in PAs, point out the existing drawbacks, propose the future research directions, and address the clinical relevance of PA proteomics data, in order to achieve our long-term goal that is use of proteomics to clarify molecular mechanisms, construct molecular networks, and discover effective biomarkers.

## Introduction

The pituitary gland, which is located in the bottom-middle of cranial cavity, functions in many biological processes. Pituitary is the most important endocrine gland of body and is composed of several kinds of cells, including corticotroph, thyrotroph, somatotroph, lactotroph, and gonadotroph, which is responsible for the secretion of corresponding hormone to regulate the endocrine system (1, 2). The endocrine functions are affected if any of the pituitary cell types goes wrong. Pituitary adenoma (PA) is the common neoplasm in pituitary gland and accounts for ca. 10% in intracranial tumors (3–5). Although most of the PAs are benign, its invasive features do exist and always comes with poor prognosis (6). The different types of PAs have different origin of cells and involve different endogenous and exogenous factors, which cause PA highly heterogeneous (3). PAs are clinically classified into functional and non-functional pituitary adenomas (FPAs and NFPAs). FPAs, commonly refer to monoclonal, originate from adrenocorticotropic hormone (ACTH) cell adenoma, thyrotropin-stimulating hormone (TSH) cell adenoma, growth hormone (GH) cell adenoma, and prolactin (PRL) cell adenoma, whereas the NFPAs commonly originate from gonadotroph cells (2, 7). NFPAs are subclassified into intact luteinizing hormone (LH^+^), follicle-stimulating hormone (FSH^+^), LH/FSH^+^, null cell, oncocytoma, silent corticotroph, and silent somatotroph subtypes (2). These differences in pathophysiological characteristics are the basis of proteomic heterogeneity in PAs. A series of proteomics strategies have been extensively used to study PAs, and the status and perspectives regarding PA proteomics were reviewed in detail by Zhan and Desiderio in the year 2004 (1). After that, significant progresses have been made in PA proteomics in the past 10 years, including sample preparation using laser-capture microdissection (LCM) (8, 9), proteomics analyses of FPA (such as prolactinoma) (10), invasive versus non-invasive PAs (6, 11), NFPA subtypes (12), serum (13, 14), protein variants/isoforms (3, 10, 15, 16), post-translational modifications (PTMs) (17–21), and protein molecular networks (22). This article reviews these progresses in PA proteomics in the past 10 years, the existing drawbacks, future perspectives, and the clinical relevance of PA proteomics data.

## Progresses in PA Proteomics

### Sample Preparation Using Laser-Capture Microdissection Coupled with Two-dimensional Liquid Chromatography

Two obvious drawbacks exist in two-dimensional gel electrophoresis (2DGE)-based quantitative comparison of proteomes between PAs and controls (1, 23). First, a PA tissue is commonly obtained from neurosurgery operation, and pituitary control tissue is commonly obtained from postmortem tissues died from other diseases or accidents. The origin of cells in PA is monoclonal, which is originated from corticotroph, somatotroph, thyrotroph, lactotroph, and gonadotroph in the pituitary gland (1). Thus, a PA tissue sample contains a relatively pure cell type, whereas postmortem pituitary control tissue sample contains multiple cell types, including corticotroph, somatotroph, thyrotroph, lactotroph, gonadotroph, and other types of cells. These differences in cell types affect the accurate comparison between PA and control tissue proteomes. Second, 2DGE with pH 3–10 IPG strip and 12% sodium dodecyl sulfate-polyacrylamide gel electrophoresis (SDS-PAGE) gel in separation of proteome is limited within a range of proteins with pH 4–8 and mass 15–150 kDa. These proteins with pI < 4 or pI > 8 and Mr > 150 kDa or Mr < 10 kDa are usually lost, which seriously impacts on the comparison between PA and control proteomes. Moreover, 2DGE has difficulty in the detection of low-abundance proteins in a proteome, which are usually associated with important physiological and pathological processes.

In order to overcome these drawbacks, LCM (24, 25) and multi-dimensional liquid chromatography (MDLC) (26) were used to analyze PA proteomes (8, 9, 27, 28). LCM can isolate, purify, and enrich tumor cells from PA tissues and the corresponding pituitary cells from control pituitaries, such as corticotroph, somatotroph, thyrotroph, lactotroph, and gonadotroph. This approach resolves the inconsistency in the origin of cells between PA and control pituitaries and achieves the accurate comparison between PA and control proteomes. Compared to 2DGE, MDLC has significant advantages in maximizing the coverage of a proteome because it is not to be limited in the detection and identification of low-abundance proteins, extremely acidic/basic proteins, extremely low/high-mass proteins, and hydrophobic proteins (26).

An FPA prolactinoma was analyzed using LCM and MDLC (8, 9, 27, 28). Prolactinoma tissue contains multiple cells, including lactotroph tumor cells, endothelial cells, fibroblasts, and other stromal cells (8). The lactotroph tumor cells were isolated and purified using immunohistochemistry-guided LCM (27, 28), and a total of 2243 unique proteins from LCM-enriched lactotroph tumor cells were identified using SDS-PAGE and 2D-LC-tandem mass spectrometry (MS/MS) (8), which form currently the biggest protein database for prolactinoma proteome. Moreover, immuno-LCM was used to enrich lactotroph cells from postmortem human pituitary tissues (9). A total of 1660 proteins from LCM-enriched lactotroph cells were identified using 2D-LC–MS/MS, which represent currently the biggest proteomic database of normal lactotroph cells. However, currently, no quantitative comparison study was carried out between LCM-enriched lactotroph tumor and normal cells to discover accurate differentially expressed proteins (DEPs) for the clarification of prolactinoma tumorigenesis.

### Significant Extension of Proteomic Reference Mapping Data in PAs and Controls

Large-scale proteomic reference mapping data are essential to discover PA-related proteins for the understanding of molecular mechanisms and discovery of reliable biomarkers of a PA. (i) For a human pituitary proteome, a total of 1449 proteins were identified using multiple gel-based technology (MGT), including in-gel isoelectric focusing (IEF)–LC–MS/MS and SDS-PAGE–LC–MS/MS (29). Most of these proteins distribute within the range of pI 3.8–12.0 (75%) and Mr 20–100 kDa (70%), and lots of low-abundance proteins were identified, including 55 protein kinases (PKAs), 45 receptors, 29 transcription factors, and 43 Ras-family proteins such as 11 G proteins. Currently, these data form the biggest protein reference map of a human pituitary proteome. (ii) For an NFPA proteome, 2DGE-based proteomics technique was used to identify 111 unique proteins in an NFPA tissue (30) and 107 unique proteins in an FSH^+^-NFPA (31), which forms the protein reference map to serve for 2DGE-based comparative proteomics analysis of NFPAs. Furthermore, the proteomic reference map of FSH^+^-NFPA contains 107 unique proteins that were involved in multiple functional systems, including receptor signal pathways, transcription and translation regulating system, bioenzyme system, oxidative stress, glutathione metabolism, gap junction, and inflammation signaling pathway, which provide a reference to in-depth analysis of the heterogeneity of NFPA proteomes (31). (iii) For a prolactinoma proteome, immno-LCM in combination with 2D-LC–MS/MS was used to identify 2243 proteins from LCM-purified lactotroph tumor cells (8) and 1660 proteins from LCM-purified normal lactotroph cells (9); these data form the biggest proteomic database for purified lactotroph tumor cells and normal cells and benefit to analyze prolactinoma tumorigenesis. Moreover, 305 proteins were identified in LCM-purified normal lactotroph cells (9) and whole pituitary control tissues (29). These extended proteomic reference data will benefit analysis of different subtypes of FPA and NFPA proteomes to discover reliable biomarkers for the understanding of molecular mechanisms, early-stage diagnosis, and therapeutic targets.

### Proteomics Analysis of Invasive Characteristics of NFPAs

Most of the PAs are benign. However, microscopic evidence shows that approximately 40% and even up to 80% of PAs have local invasion to surrounding structures (32–35), which causes difficulty in complete removal of a tumor through neurosurgery, increases the risks of complications and poor therapeutic outcome, and results in a need of adjuvant radiotherapy or medication after neurosurgery. Therefore, the invasive characteristics of PAs are a challenging clinical problem. Clarification of molecular mechanisms and discovery of protein biomarkers regarding invasiveness of PAs are necessary to better manage an invasive PA patient (36). Quantitative transcriptomics study revealed 346 differentially expressed genes (DEGs) (233 upregulated and 113 downregulated) in invasive relative to non-invasive NFPAs (36). A 2DGE-based comparative proteomics discovered 30 DEPs between invasive and non-invasive PAs (11). Furthermore, 2DGE proteomic comparison between 4 invasive and 4 non-invasive NFPAs (6) identified 57 DEPs (30 upregulated and 27 downregulated) from 103 differential 2D gel spots (64 upregulated and 39 downregulated), and these DEPs were involved in several invasiveness-related pathway networks, including oxidative stress, mitochondrial dysfunction, MAPK-signaling abnormality, ketogenesis and ketolysis, TR/RXR activation, proteolysis abnormality, CDK5 signaling abnormality, and amyloid processing. These findings provide important clues to insight into the molecular mechanisms of invasiveness of an NFPA and discovery of reliable protein biomarkers to guide adjuvant treatment for post-neurosurgery invasive NFPAs.

### Proteomics Analysis of Heterogeneity of NFPAs

Non-functional pituitary adenoma is a highly heterogeneous neoplasm with different origin of cells and multiple hormone-expressed subtypes, including intact gonadotroph with LH/FSH^+^ (40–79%), silent somatotroph with GH expression (3%), silent corticotroph with ACTH expression (8%), null cell without hormone expression (17%), and oncocytoma without hormone expression (6%) (3, 37, 38). The intact gonadotroph may lead to several hormone-expressed subtypes of NFPAs, including LH^+^, FSH^+^, or LH/FSH^+^. The interesting thing is that silent hormone expression associates the invasiveness of an NFPA. For example, ACTH^+^-NFPA was more invasive and had higher recurrence rate than ACTH^−^-NFPA (39). The imbalance of ERα and ERβ expressions is involved in the pathological process and tumor biological behavior, such as invasiveness of an NFPA (40), and the ERα/ERβ ratio was positively related to invasive degree of an NFPA. Thereby, it is necessary to investigate proteomic heterogeneity among NFPA subtypes to discover the common and specific biomarkers for the accurate understanding of molecular mechanisms, early-stage diagnosis, and prognostic assessment and the therapeutic targets toward personalized and precise medicine. 2DGE-based comparative proteomics was used to identify 76 DEPs in NF^−^, LH^+^, FSH^+^, and LH/FSH^+^ (NF^−^ means NFPA that had negative immunohistochemical stains for LH, FSH, ACTH, GH, PRL, and TSH) relative to pituitary controls (12). Of them, 44 DEPs (10 upregulated and 34 downregulated) were common to these 4 NFPA subtypes. The rest of DEPs were common or specific to some of the four NFPA subtypes. Four pathway–network systems, including mitochondrial dysfunction, oxidative stress, MAPK-signaling abnormality, and cell-cycle dysregulation, were common to four NFPA subtypes. However, many differences existed in each pathway–network system among four NFPA subtypes, including different expression levels of each protein node, different protein profiles, and different pathway–network profiles (12). These data provided a heterogeneous profile of human NFPA proteomes that would benefit in-depth clarification of NFPA heterogeneity, molecular mechanisms of NFPAs, and discovery of effective biomarkers for early-stage diagnosis, therapy, and prognosis for NFPA personalized and precise medicine practice.

### Proteomics Analysis of Protein Variants/Isoforms and PTMs of PAs

The process of a gene-coded protein involves multiple post-transcriptional/post-translational regulations, including splicing, modifications, translocation, and spatial conformation (41–43). Protein variants/isoforms are mainly derived from splicing and PTMs. Protein variants/isoforms and PTMs are very important aspects in a proteome because they function in multiple physiological and pathological processes and would be biomarkers and therapeutic targets (44). Currently, exploration of protein variants/isoforms and PTMs is much insufficient in the width and depth in a PA proteome. By far, GH (or PRL) variants/isoforms in a human pituitary proteome have been documented (3, 10, 15, 16). PTMs regarding phosphorylation and nitration in PA and control proteomes have been reported (17–21).

Growth hormone is a very important hormone in the body. Abnormal secretions of GH are associated with many GH-related diseases, such as dwarfism, gigantism, acromegalia, and adenoma. The synthesis and release of human GH (hGH) are regulated under a series of factors (15). The hGH/IGF1 axis plays an important role in stimulating body growth (45), whereas some PTMs, such as phosphorylation, involve signal transduction pathway of hGH/IGF1 axis (46). Four hGH isoforms have been found in some GH-producing adenomas. Study (47) demonstrates that extensively molecular heterogeneity existed in hGH because of alternative splicing, different PTMs, and proteolytic processing and that circulating hGH composites of various proportions among three non-22-kDa GH isoforms 2–4 in various GH-related diseases. 2DGE-based proteomics found that 24 2D gel spots contained hGH, with significantly different expression proportions of these isoforms, namely, isoform 1 (87.5%) > isoform 2 (8.1%) > isoform 3 (3.3%) > isoform 4 (1.1%) (15). Moreover, three phosphorylation sites (Ser-77, Ser-176, and Ser-132) and a deamidation at Asn-178 were identified in the hGH amino acid sequence. Also, many studies demonstrate the ratio of non-22 kDa GH isoform might be a marker for normal and abnormal growth (48), and the evaluation of these GH isoforms could be a clinical monitoring for acromegalic patients (49). A 2DGE-based comparative proteomics (21) found at least 17 GH isoforms and 6 PRL isoforms in human pituitary proteomes, and not all these GH and PRL isoforms were associated with PAs.

Phosphorylation in a protein is an important PTM, which is involved in the regulation of multiple critical biological functions, such as protein–molecule (DNA, RNA, and protein) interactions, enzyme activity, protein trafficking, protein intracellular localization, and protein degradation, and is extensively associated with different diseases, such as cancer, inflammation, and neurodegenerative diseases (17). Phosphorylation occurs in Ser, Thr, and Tyr residues in a protein and is dynamically and reversibly regulated with PKAs and phosphatases that are the common drug targets, and much attention has been paid to protein phosphorylation event (50). Protein phosphorylation analysis was carried out in pituitary control tissues, which identified a total of 28 phosphoproteins from 2 different studies (17, 18), including 8 phosphorylation sites in 6 pituitary phosphoproteins that were characterized using immobilized metal ion affinity chromatography (IMAC)-based LC–MS/MS from unseparated digested pituitary protein mixture (18) and 50 phosphorylation sites in 26 pituitary phosphoproteins (73 phosphopeptides) that were characterized using a modified strategy-in-gel IEF–LC–MS/MS from digested pituitary protein mixture (17). These data expanded human pituitary phosphoprotein database. However, quantitative phosphoproteomics has not been performed between PAs and controls to discover PA-related phosphoproteins and phosphosites for in-depth understanding of biological significance of phosphorylation in PAs.

Tyrosine nitration in a protein is another important PTM that is mainly derived from *in vivo* peroxynitrite (ONOO^−^) pathway, myeloperoxidase pathway, and metalloperoxidase reaction pathway (19, 51, 52). Nitration decreases the electron density of a phenolic ring of tyrosine residue to affect the affinity ability between protein–protein interactions, such as enzyme–substrate, receptor–ligand, and antigen–antibody, and is extensively associated with different pathophysiological processes, such as cancer, neurodegenerative disease, and inflammation (19). Nitroproteomics was performed in pituitary controls (20, 53) and adenomas (21). Eight nitroproteins and eight nitrotyrosine sites were characterized from a pituitary control tissue using 2DGE-based nitrotyrosine Western blot coupled with MS/MS (20, 53), including cGMP-dependent PKA 2, stanniocalcin 1, synaptosomal-associated protein, actin, immunoglobulin α Fc receptor, mitochondrial co-chaperone protein HscB, progestin and adipoQ receptor family member III, and proteasome subunit α type 2. Nine nitroproteins and 10 nitrotyrosine sites were characterized from a PA tissue using nitrotyrosine affinity column (NATC)-based MALDI–MS/MS (21), including sphingosine-1-phosphate lyase 1, zinc finger protein 432, cAMP-dependent PKA type I-β regulatory subunit, Rho-GTPase-activating protein 5, leukocyte immunoglobulin-like receptor subfamily A member 4, centaurin-β 1, proteasome subunit α type 2 interleukin 1 family member 6, and rhophilin 2. Moreover, three nitroprotein–protein complexes were identified, including nitrated proteasome–ubiquitin complex, nitrated β-subunit of cAMP-dependent PKA complex, and nitrated interleukin 1 family member 6-interleukin 1 receptor–interleukin 1 receptor-associated kinase-like 2 (IL1F6–IL1R–IRAK2) (21). Moreover, protein domain/motif analysis found that most nitrotyrosine sites occurred within important protein domains and motifs (21), and pathway–network analysis found that these nitroproteins function in important signaling pathway-network systems (22), which demonstrated that tyrosine nitration might play important roles in the formation of a human PA. However, quantitative nitroproteomics has not been performed between PAs and controls to discover PA-related nitroproteins and nitrotyrosine sites for in-depth understanding of biological significance of tyrosine nitration in PAs.

### Serum Proteomics and Peptidomics in NFPAs

No biomarker is used to early diagnose an NFPA, and NFPA is usually diagnosed exclusively using CT or MRI after appearance of compression symptoms or sometimes by chance (13). Serum is an important window to predict, early diagnose, and prognosis assess a PA. One study (13) analyzed 34 sera from NFPA patients and 34 sera from age-/sex-matched healthy controls using surface-enhanced laser desorption/ionization time-of-flight mass spectrometry (SELDI-TOF-MS) and identified 9 serum DEPs (7 upregulated and 2 downregulated) with both 82.4% sensitivity and specificity in discrimination of NFPAs from controls. Another study (14) quantitatively compared 65 sera from PAs and 90 sera from healthy controls using proteomic fingerprint technology that was magnetic beads-based MALDI-TOF-MS and identified 42 *m/z* peaks that were significantly related to PAs (14). A three-biomarker diagnostic model was established with 90.0% sensitivity and 88.3% specificity in discrimination of human PAs from controls, which provided a powerful and reliable method to diagnose a PA (14).

### Protein Antibody Array Analysis of PAs

Protein antibody array is an effective method to detect alterations of known proteins in PAs and easily translated to biomarkers for prediction, diagnosis, and prognostic assessment of PAs. A protein antibody array with 1005 monoclonal antibodies (54) identified 316 proteins in GH-, ACTH-, and PRL-secreting FPAs, and in NFPAs. Also, a set of DEPs were identified and confirmed using immunohistochemistry and Western blotting.

### Proteomic Data-Based Pathway Networks in NFPAs

Each protein functions in the corresponding pathway–network system to exert its biological roles. The IPA pathway analysis is a widely used pathway analysis system to identify statistically significant signaling pathway networks from a set of qualitative and quantitative proteomic data (22), including 111 proteins from a human NFPA tissue, 56 DEPs from human NFPA tissues and from prolactinoma tissues, 9 nitroproteins and 3 nitroprotein–protein complexes from a human NFPA tissue, and 8 nitroproteins from a pituitary control tissue. Comprehensive analysis of these pathway networks revealed four signaling pathway–network systems in NFPAs, including mitochondrial dysfunction, oxidative stress, cell-cycle dysregulation, and MAPK-signaling abnormality (22). These data provided potential biomarkers and contributed to the development of new efficacious treatment targets for PAs.

## Clinical Relevance of PA Proteomics Data

A great methodological progress of PA proteomics and use of proteomics approach to study pathophysiological characteristics of PAs have been reviewed, as described above. Here, the clinical relevance of PA proteomics data (3, 6, 10–12, 55) is further addressed in the following aspects.

(i) Insights into molecular mechanisms of PAs. Proteomics offers a unique insight into molecular mechanisms of pituitary tumorigenesis at the level of proteome. Our multiple comparative proteomics studies have confirmed our initial hypothesis that the proteome differs between PAs and controls (3, 10), between invasive and non-invasive PAs (6, 11), and among different types of PAs (12); many DEPs and molecular networks were related to transcriptomics data (3, 10, 36, 56) and to the corresponding biological systems (6, 12, 22). These DEPs and molecular networks provided a basis to determine alterations in multiple protein systems that contribute to pituitary tumorigenesis. A set of DEP data (3) and DEG data (56) were correlated and obtained many protein systems that connect some critical aspects of PA formation (Table 1). First, the alterations in endocrine hormone levels are the significantly clinical characteristics of PAs. Many pituitary hormones were found to change, including GH, TSH, PRL, LH, and FSH. The synthesis and secretion of each hormone are the important function of pituitary organ and are closely regulated by various signaling pathways and factors. The cGMP-dependent PKA and nitric oxide are taken, for example, that significantly regulates water-soluble hormones. Proteomics study (20) discovered that the cGMP-dependent PKA 2 was nitrated in human pituitary tissues, which indicates that the nitration of cGMP-dependent PKA might be involved in the signal processing of water-soluble hormones. Second, the alterations in signal transduction systems significantly participate in the pathogenesis of PAs. Many signal pathways were found by omics data to change, including G proteins, cytokine receptors (IFG, IL, EGF, TGF, and IFN), and some signal system-regulated enzymes (MAPK4, phospholipase A2-IB, and cAMP-dependent PKA). G proteins have been confirmed to play an important role in PAs (57), and FGF family members are involved in pituitary pathogenesis (58). FGF receptor genes mediate FGF signal pathways, and these genes encode receptor tyrosine kinases. Our proteomic DEP data clearly have a Tyr PKA. In addition, phosphorylation is significantly involved in signal pathways, and a Ser/Thr protein phosphatase 2A was found in our proteomics data (3). Third, oxidative/nitrative stresses are significantly associated with multiple cancer pathogenesis. Proteomics DEP data (3) demonstrate that some reactive-oxygen species (ROS)-related proteins are changed in PAs, including cytochrome P450, heat shock protein, GST, and phospholipid-hydroperoxide glutathione peroxidase. Fourth, DNA methylation was found to participate in pituitary tumorigenesis (58); proteomics study (3) revealed a methylation-related DEP (Table 1), and three methylation-related DEGs were found by transcriptomics study (56). Fifth, oncogene activation in PAs (such as c-Myc) was found (59); protooncogene-related DEPs (3) and -related DEGs were found (56) (Table 1). Furthermore, multiple proteomics data-based molecular network studies revealed four important pathway–network systems that were altered in NFPAs, including mitochondrial dysfunction, oxidative stress, cell-cycle dysregulation, and MAPK-signaling system abnormality (22); and the last three pathway–network systems were involved in protein tyrosine nitration, for example, NRF2-mediated oxidative stress response, cell-cycle G2/M DNA damage checkpoint regulation, and p38 MAPK signaling were identified from PA nitroproteomic data (22). It shows that tyrosine nitration is involved in pituitary tumorigenesis. Finally, invasiveness of PAs is a challenging clinical problem. Fifty-seven DEPs (30 upregulated and 27 downregulated) were found by proteomic study to associate the invasive features of PAs (6), and invasiveness-related pathway networks were found including MAPK-signaling abnormality, mitochondrial dysfunction, oxidative stress, proteolysis abnormality, ketogenesis and ketolysis, TR/RXR activation, CDK5 signaling abnormality, and amyloid processing (6). These data demonstrate an overview of multiple protein systems that are involved in the pathogenesis of PAs.
(ii) Discovery of potential biomarkers and therapeutic targets for PAs. DEPs and DEGs (Table 1) (3, 56) and the consistently altered DEPs and DEGs in the levels of protein and mRNA (Table 2) (3, 10), which were identified with proteomics and transcriptomics studies, are a part of our novel source of candidate biomarkers and therapeutic targets. Secretagogin was taken, for example (60). Secretagogin is a type of Langerhans-specific Ca^2+^-binding protein (61) and is highly expressed in the secretory neurons of the anterior pituitary (62). Secretagogin can inhibit the transcription of excitatory amino acid substance P (SP) to lower the growth rate of RIN-5F tumor cells (61), which is consistent with Desiderio’s hypothesis that an imbalance between inhibitory (opioid) and excitatory (tachykinin) neuropeptidergic systems might contribute to the pathogenesis of a pituitary macroadenoma (63–67). Proteomics and transcriptomics analyses (60) revealed a significant downregulation of secretagogin in a set of human NFPAs, and its protein expression was significantly related to its mRNA expression. These data demonstrate that secretagogin might be a biomarker for PAs. Moreover, other candidate biomarkers, such as G proteins, tissue transglutaminase, isocitrate dehydrogenase (NADP) cytoplasmic (IDH1), calreticulin, cytokine receptors, and ROS-related proteins (Tables 1 and 2), need to be further studied for potential biomarkers and therapeutic targets.
(iii) Pituitary hormone variants/isoforms and PTMs in human PAs. Protein variants/isoforms result from splicing, PTMs, etc. The alterations in neuroendocrine hormone levels are the important characteristics of PAs. Proteomics studies demonstrate that human pituitary hormones had multiple variants/isoforms. Not all of variants/isoforms were associated with PAs (3). First, GH variants/isoforms and PTMs. Somatotropin was significantly downregulated at the mRNA and protein levels in NFPAs (3) and in prolactinomas (10), which was consistent with their monoclonal composition in origin (68, 69) – a NFPA generated from gonadotrophs and a prolactinoma from lactotrophs, whereas GH receptor gene was unchanged. These data showed that GH hyposecretion in NFPAs results from the hypoexpression of the GH gene. However, the interesting finding is that multiple GH variants/isoforms (17 spots contain somatotropin) were detected (3, 10); these data cannot be interpreted by transcriptomics method. The downregulated ratio of different GH isoforms was different in each cell type of PA relative to controls. These data suggested that the proportion of different GH isoforms changed in each cell type adenoma compared to controls. Other researchers showed that the proportion of the circulating GH isoforms significantly changed in PAs and other pituitary diseases (48, 49). The proportional change in the different GH isoforms might have an important value in the clinical evaluation of human PAs. Recently, we found that GH isoforms were derived from various splicing variants and PTMs, including phosphorylation. The phosphorylation of endogenous GH in the human pituitary (18) provided us with new insights into the mechanisms of GH that participates in the neuroendocrine signal pathways. Second, PRL variants/isoforms and PTMs. PRL is another important pituitary hormone. Proteomics detected six PRL variants/isoforms (3, 10). Similar to GH, each PRL variant/isoform was downregulated in each cell type of NFPA, with a different downregulated ratio relative to controls. There was no significant expression change in the PRL gene at the mRNA and protein levels in the prolactinomas relative to controls, which is consistent with a prolactinoma’s monoclonal composition in origin. However, the proportion of six PRL variants/isoforms was changed in prolactinomas compared to controls. Other researchers showed that glycosylation was an important PRL modification (70) that produces different variants/isoforms; glycosylation of human PRL might downregulate its hormone bioactivity and promote its metabolic clearance (71). Other studies showed that the main variant of PRL was non-glycosylated form of PRL in human prolactinomas (72), which is consistent with our result that some PRL isoforms (10) were downregulated in prolactinomas relative to controls. Thus, we speculated that PRL variants/isoforms in these downregulated spots were possibly glycosylated PRL. Therefore, our detailed study of different variants/isoforms and PTMs of GH and PRL could lead to new insights into the clinical importance of pituitary hormones in a PA. The ratio of the change of each variant/isoform in human pituitary tissue has value for clinical research. However, one must realize that studies regarding protein variants/isoforms and PTMs are much insufficient in the width and depth in PAs, much more efforts should be made to insight into this area in a PA proteome because protein variants/isoforms and PTMs play very essential roles in tumor pathogenesis and could be potential biomarkers and therapeutic targets (44).
(iv) Use of proteomic variations for personalized and precise medicine practice of PAs. We addressed in detail how to use proteomic variations for prediction, prevention, and personalized medicine in NFPAs (73) and use the integrated omics data for personalized and precise treatment for PAs with view point of multi-parameter systematic strategy (74, 75). Although there is a long distance to reach the extreme goal of personalized and precise medicine of PAs, proteomic variations in combination with integrated omic variations and the development of novel technologies offer the great promise to achieve the goal (73–75).

**Table 1:** **Biological systems that operate in pituitary adenomas**.

Systems	DEPs (*n* = 56) (3)	DEGs (*n* = 128) (56)
**(A) Hormones**		
(1) GH	Somatotropin	GHRH receptor
(2) TSH		TSH β
(3) PRL	Prolactin	PRL
(4) LH		LH β
(5) FSH		FSH β
**(B) Signal transduction**		
(1) G proteins^a^	G_0_ subunits 1, 2^a^	G protein
		G-binding protein
		Regulator of G-protein signaling 16
		Regulator of G-protein bind signaling 2, 5
		Rho-GTPase-activating protein 5
(2) Cytokine receptors		
(a) IGF	IGF binding	IGF
		IGF binding
		IGF binding 5
		IGF binding 3
		Protease Ser 11 IGF binding
(b) IL	Splice isoform IL-15	
(c) EGF		EGF-containing fibulin ECM protein 1
(d) TGF		TGFα receptor III
(e) IFN		Interferon-induced protein 56
(3) Signal-system enzymes		MAPK 4
		Phospholipase A2-IB
		Protein kinase cAMP-dependent β
(4) Retinoic acid		Cellular retinoic acid-binding protein 2
(5) Phosphorylation	Ser/Thr protein phosphatase 2A	
(6) Others	Rab GDP dissociation inhibitor α	SH3-domain GRB2-like 2
		*XIST* nuclear receptor 1, 3
**(C) Reactive-oxygen species**		
(1) Cytochrome P450		Cytochrome P450, III A
(2) HSP	HSP27	HSP 105 kDa
(3) GST	GSTμ-2	
(4) Peroxidase	Phospholipid-hydroperoxide glutathione peroxidase	
**(D) DNA/mRNA methylation**	^6^*N*-adenosine methyltransferase^a^	RNA-binding protein 2
		Eukaryotic translation initiation factor 3–5
		U4/U6-associated RNA splicing factor
**(E) Tumor genes**		
(1) Protooncogenes^a^	Protooncogene Tyr protein kinase FYN^a^	
	L-myc-1 protooncogene protein	
(2) Oncogenes		Neuroblastoma overexpressed gene
(3) Tumor suppressor genes	Tumor rejection antigen (endoplasmin)	
**(F) Excitatory (tachykinins)**	Secretagogin	Reticulocalbin l, EF-hand calcium-binding domain
		Peptidylglycine α-amidating monooxygenase
**(G) Energy**	ATP synthase, mitochondrial	ATPase
	ATP-binding protein	
**(H) Immune system**	Ig κ, λ	IgG
		Pre-B cell leukemia transcription factor 3
		CD58
		MHC class 1
**(I) Intermediate filaments**	Vimentin	Vimentin
**(J) Transcription factors**		Pit 1
		Basic transcription factor 3
**(K) Cell cycle (G_1_-S-G_2_-M)**		Death-associated protein
		Folate receptor C1

*^a^Publication data indicated that G protein (57), methylation (58), c-myc (59), PTTG, gsp, ccnd1 (59), FGF (76), Men-1, Prop-1, c-jun, Fos, and angiogenesis were related to pituitary adenomas*.

*DEPs, differentially expressed proteins; DEGs, differentially expressed genes*.

*Modified from Zhan et al. (55), with permission from Elsevier Science Publisher, copyright 2007*.

**Table 2:** **Genes that altered consistently in the levels of protein and mRNA with comparison of proteomics data and transcriptomic data**.

DEPs				DEGs			
Swiss-prot	Protein name	NF	PRL	Genbank	Gene name	NF	PRL
O76038	Secretagogin (SCGN)	−	− (weak)	NM_006998	SCGN: secretagogin	−	
P16520	Guanine nucleotide-binding protein G(I)/G(S)/G(T) β-subunit 3	+		M31328	Guanine nucleotide-binding protein (G protein), β polypeptide	+	
P21980	Tissue transglutaminase (TGM2)	−	−	NM_004613	TGM2: transglutaminase 2	−	−
O75874	Isocitrate dehydrogenase (NADP) cytoplasmic (IDH1)	+	+ (weak)	NM_005896	IDH1: isocitrate dehydrogenase (NADP+), soluble	+	
P27797	Calreticulin precursor	+		AI807225	KDEL endoplasmic reticulum (ER) protein	+	
P01241	Somatotropin (GH1)	−	−	NM_000515	GH1: growth hormone 1	−	−
				NM_002059	GH2: growth hormone 2	−	−
				NM_000823	GHRHR: growth hormone releasing hormone receptor	−	−
P01236	Prolactin (PRL)	−	±	NM_000948	PRL: prolactin	−	±
P29373	Cellular retinoic acid-binding protein II	+		M97815	Cellular retinoic acid-binding protein 2	+	
gi1066765	Hemoglobin β chain (HBB)	−	−	NM_000518	HBB: hemoglobin β	−	−
				NM_000519	HBD: hemoglobin δ	−	

*The bracket after the protein name refers to its corresponding gene name*.

*NF, non-functional pituitary adenoma; PRL, hyperprolactinoma; +, upregulated in pituitary adenomas relative to controls; −, downregulated in pituitary adenomas relative to controls*.

*Reproduced from Zhan and Desiderio (1), with permission from Wiley-VCH, copyright 2005*.

## Future Perspectives in PA Proteomics

Although a great progress has been achieved in human PA proteomics, a series of unsolved issues are still existing in the following aspects. (i) High-throughput quantitative proteomics based on LCM-purified PA and control cells. LCM can effectively purify the PA cells and the corresponding pituitary cells for protein mapping analysis (8, 9) and minimize the inconsistency in cell types between PA tissues and postmortem pituitary control tissues. However, high-throughput quantitative proteomics strategies, such as isobaric tags for relative and absolute quantification (iTRAQ) (77, 78), label-free strategies (79, 80), or sequential window acquisition of all theoretical spectra (SWATH) (81, 82), have not been used to determine DEPs between LCM-purified PA cells and the corresponding control cells (somatotroph, corticotroph, lactrotroph, gonadotroph, and tyrotroph) for more accurate understanding of molecular mechanisms of different cell types of PAs and discovery of reliable biomarkers. (ii) PTM-quantitative proteomics in PAs. PTMs are very important aspects in a proteome (44). However, PTM proteomics studies in PAs are much insufficient in depth and width. Currently, only 28 phosphoproteins from pituitary control tissues (17, 18), 8 nitroproteins from a pituitary control tissue (20, 53), and 9 nitroproteins from an NFPA tissue (21) have been identified. PTM-quantitative proteomics (such as iTRAQ- and label-free strategies) has not been performed to discover PA-related PTMs, such as phosphorylation (83, 84), nitration (19, 52), acetylation (85, 86), glycosylation (87, 88), methylation (89), ubiquitylation (90), sumoylation (91), and succinylation (92); however, it is a very promising area in the field of PA proteomics. (iii) Structural proteomics of PAs. Three-dimensional (3D) structure of a protein determines its biological significance in a PA biological system (52). If 3D structure of a key PA protein can be reconstructed with X-ray crystallography data, then it would easily interpret its biological roles and is possible to design a small-molecule drug toward that 3D structure for the development of effective therapy strategy for PAs. (iv) Body fluid proteomics/peptidomics of PAs. PA is a whole-body disease with multiple factors, multiple processes, and multiple consequences (74). Body fluid, such as CSF and serum, is the window for prediction, diagnosis, and prognostic assessment of a PA (73). Body fluid proteomics/peptidomics is barely performed and is very promising research direction for PAs, especially NFPAs. (v) Omics data-based systems biology and molecular networks. PA is a complicated whole-body disease with alterations in multiple molecules in the levels of genome, transcriptome, proteome, and metabolome; and these molecules are mutually associated within a molecular network system (75). Multiple omics data-based systems biology and molecular network analyses might discover key molecular networks that are common or specific to different types of PAs and benefit discovery of effective and reliable biomarkers. (vi) Invasive mechanisms and biomarkers of FPAs and NFPAs. Proteomics studies regarding invasive characteristics of PAs analyzed only invasive and non-invasive NFPAs with 2DGE-quantiative proteomics (6, 11). Because of 2DGE disadvantages, further non-gel high-throughput quantitative proteomics is needed (26). Moreover, this type of study should expand to invasive and non-invase FPAs. The clarification of invasive mechanisms and determination of biomarkers might benefit management of invasive PAs. (vii) Heterogeneity of NFPAs. NFPA is highly heterogeneous. Current proteomics study focused only on NF^−^-, LH^+^-, FSH^+^-, and LH/FSH^+^-NFPA subtypes with 2DGE-quantitative proteomics (12). It should expand to other NFPA subtypes, such as silent corticotroph adenoma and silent somatotroph adenoma. Moreover, current proteomics studies of PAs mainly focused on NFPAs (1, 3, 6, 12), but barely analyzed FPAs (10). Therefore, in future, more efforts toward research on FPAs with proteomics strategies are required. The clarification of proteomic variations in different types of NFPAs and FPAs would benefit complete understanding of molecular mechanisms and discovery of biomarkers that are common and specific to different types of PAs and really realize personalized and precise medicine practice of PAs.

## Conclusion

The use of proteomics to clarify molecular mechanisms of PAs and discover reliable biomarkers for prediction, prevention, and personalized treatment for PAs is our long-term goal. A great progress has been achieved in the field of PA proteomics in the past 10 years, including sample preparation with LCM and 2D-LC–MS/MS (8, 9), significant extension of proteomic reference mapping data in PAs and controls (8, 9, 29–31), proteomics analyses of invasive characteristics (6, 11), tumor heterogeneity (12), protein variants/isoforms (3, 10, 15, 16) and PTMs (17–21), and serum proteomics and peptidomics (13, 14) of PAs, protein antibody array analysis of PAs (54), and proteomic data-based pathway networks in NFPAs (22). These new progresses of pituitary proteomics are encouraging and provide new promise for insight into pathophysiological system of PAs. However, there are many aspects that have not been resolved and are worth studying in-depth including high-throughput quantitative proteomics based on LCM-enriched pituitary cells, PTM-quantitative proteomics, structural proteomics, body fluid proteomics and peptidomics, omics data-based integrative molecular networks, and proteomic characteristics of invasiveness and heterogeneity of PAs. Furthermore, one must realize that, although clinical relevance of PA proteomics data was discussed in this article, the real functional analysis regarding most of these interesting proteins from PA proteomics studies has not been carried out in the PA biological systems, which is very important aspect in the post-proteomics and must be strengthened in future. Resolving all these aspects will benefit systematical and complete understanding of PA pathogenesis and management of PAs toward personalized and precise medicine practice.

## Author Contributions

XZ conceived of the concept and idea of this article, collected reference, wrote and revised the entire manuscript, was responsible for the corresponding work of the manuscript. XW collected references and drafted partial manuscript. TC participated in revision and edition of the manuscript. All authors read and approved the final manuscript.

## Conflict of Interest Statement

The authors declare that the research was conducted in the absence of any commercial or financial relationships that could be construed as a potential conflict of interest.

## Abbreviations

2D, two-dimension; 2DGE, two-dimensional gel electrophoresis; ACTH, adrenocorticotropic hormone; CSF, cerebrospinal fluid; DEG, differentially expressed gene; DEP, differentially expressed protein; FPA, functional pituitary adenoma; FSH, follicle-stimulating hormone; GH, growth hormone; IEF, isoelectric focusing; IL1, interleukin 1; IMAC, immobilized metal ion affinity chromatography; IPA, ingenuity pathway analysis; LC, liquid chromatography; LCM, laser-capture microdissection; LH, luteinizing hormone; MALDI, matrix-assisted laser desorption ionization; MDLC, multi-dimensional liquid chromatography; MGT, multiple gel-based technologies; MS/MS, tandem mass spectrometry; MS, mass spectrometry; NFPA, non-functional pituitary adenoma; NTAC, nitrotyrosine affinity column; PA, pituitary adenoma; PKA, cAMP-dependent protein kinase; PPPM, predictive, preventive and personalized medicine; PRL, prolactin; PTM, post-translational modification; SDS-PAGE, sodium dodecyl sulfate-polyacrylamide gel electrophoresis; SELDI, surface-enhanced laser desorption/ionization; TOF, time-of-flight; TSH, thyrotropin-stimulating hormone or thyrotropin.
